# Novel early‐onset Alzheimer‐associated genes influence risk through dysregulation of glutamate, immune activation, and intracell signaling pathways

**DOI:** 10.1002/alz70855_107379

**Published:** 2025-12-24

**Authors:** Joseph Bradley, Daniel Western, Ciyang Wang, Eder Lucio da Fonseca, Achal Neupane, Jiji T. Kurup, Nicholas R. Ray, Melissa Jean‐Francois, Kristy Bergmann, John P. Budde, Eden R. Martin, Margaret Pericak‐Vance, Michael L Cuccaro, Adam C. Naj, Brian W Kunkle, Alzheimer's Disease Genetics Consortium, Knight Alzheimer Disease Research Center (Knight ADRC), Gerald D. Schellenberg, Victoria Fernández, Jonathan L Haines, John C. Morris, David M. Holtzman, Richard J. Perrin, Carlos Cruchaga, Christiane Reitz, Gary W Beecham

**Affiliations:** ^1^ Department of Psychiatry, Washington University School of Medicine, St. Louis, MO, USA; ^2^ NeuroGenomics and Informatics Center, Washington University School of Medicine, St. Louis, MO, USA; ^3^ John P. Hussman Institute for Human Genomics, Miller School of Medicine, University of Miami, Miami, FL, USA; ^4^ NeuroGenomics and Informtics, Washington University School of Medicine, St. Louis, MO, USA; ^5^ Columbia University, The Taub Institute for Research on Alzheimer's Disease and the Aging Brain, The Gertrude H. Sergievsky Center and Departments of Neurology and Epidemiology, New York, NY, USA; ^6^ Taub Institute for Research on Alzheimer's Disease and the Aging Brain, Columbia University, New York, NY, USA; ^7^ John P. Hussman Institute for Human Genomics, University of Miami Miller School of Medicine, Miami, FL, USA; ^8^ NeuroGenomics and Informatics Center, Washington University, St. Louis, MO, USA; ^9^ Department of Psychiatry, Washington University, St. Louis, MO, USA; ^10^ Dr. John T. Macdonald Foundation Department of Human Genetics, University of Miami Miller School of Medicine, Miami, FL, USA; ^11^ Penn Neurodegeneration Genomics Center, University of Pennsylvania Perelman School of Medicine, Philadelphia, PA, USA; ^12^ University of Pennsylvania, Perelman School of Medicine, Department of Biostatistics and Epidemiology/Center for Clinical Epidemiology and Biostatistics, Philadelphia, PA, USA; ^13^ University of Pennsylvania School of Medicine, Philadelphia, PA, USA; ^14^ Knight Alzheimer's Disease Research Center, Washington University, St. Louis, MO, USA; ^15^ Penn Neurodegeneration Genomics Center, Department of Pathology and Laboratory Medicine, University of Pennsylvania Perelman School of Medicine, Philadelphia, PA, USA; ^16^ Ace Alzheimer Center Barcelona‐Universitat Internacional de Catalunya, Barcelona, Barcelona, Spain; ^17^ Department of Population and Quantitative Health Sciences, Case Western Reserve University School of Medicine, Cleveland, OH, USA; ^18^ Department of Population & Quantitative Health Sciences, School of Medicine, Case Western Reserve University, Cleveland, OH, USA; ^19^ Department of Neurology, Washington University School of Medicine, St. Louis, MO, USA; ^20^ Knight Alzheimer Disease Research Center, Washington University School of Medicine, St. Louis, MO, USA; ^21^ Knight Alzheimer Disease Research Center, St. Louis, MO, USA; ^22^ Department of Pathology and Immunology, Washington University School of Medicine, St. Louis, MO, USA; ^23^ Department of Neurology, Washington University in St. Louis School of Medicine, St. Louis, MO, USA; ^24^ Washington University School of Medicine, St. Louis, MO, USA; ^25^ Gertrude H. Sergievsky Center, Taub Institute for Research on the Aging Brain, Departments of Neurology, Psychiatry, and Epidemiology, College of Physicians and Surgeons, Columbia University, New York, NY, USA; ^26^ Department of Biostatistics and Data Science, Wake Forest School University of Medicine, Winston‐Salem, NC, USA

## Abstract

**Background:**

Alzheimer Disease (AD) is a highly polygenic dementia that presents with relatively earlier onset (≤65yo; EOAD) in about 5% of cases. Around 90% of these EOAD cases remain unexplained by pathogenic mutations.

**Method:**

We used data from EOAD cases ≤ 70 and controls > 70 to ensure they would not develop AD. genome‐wide association study (GWAS) and trans‐ancestry meta‐analysis were performed for non‐Hispanic European (EUR, N_Case_=6,282, N_Control_=13,386), African (AFR N_Case_=782, N_Control_=3,663) and East Asian (ASN: N_Case_=375, N_Control_=838 CO) ancestries. We performed QTL mapping and *in‐silico* annotation to identify the functional gene for each genome‐wide loci, and PRS and LDSC to compare the genetic architecture of EOAD vs LOAD

**Result:**

We identified a total of 15 genome‐wide significant loci, 7 already reported to be associated with LOAD and 8 novel loci. Of the novel loci: six were found in the ancestry‐specific analyses and two in the trans‐ancestry analysis. By integrating gene‐based analysis, eQTL, pQTL and functional annotations, we nominate four novel functional genes (*CDH12, FOLH1, ALG10B* and *LRRC25*) for four loci that are involved in microglia activation, glutamate production, and signaling pathways. LOAD‐derived PRS showed a strong association with EOAD (R^2^=0.170, *p* <10^‐300^, and R^2^=0.048, *p* = 1.20×10^‐143^: for PRS with and without *APOE*). LDSC found strong overall genetic covariance between EOAD and LOAD (rg=0.791, *p* = 6.80×10^‐12^)

**Conclusion:**

These results indicate that EOAD, although sharing many genes with LOAD, harbors unique genes and pathways that could be used to create better prediction models or target identification for this type of AD.

